# Comparison of Transgenerational Neurotoxicity between Pristine and Amino-Modified Nanoplastics in *C. elegans*

**DOI:** 10.3390/toxics12080555

**Published:** 2024-07-30

**Authors:** Mingxuan Song, Qinli Ruan, Dayong Wang

**Affiliations:** 1 School of Medicine, Nanjing University of Chinese Medicine, Nanjing 210023, China; 2 Medical School, Southeast University, Nanjing 210009, China; 3 Shenzhen Ruipuxun Academy for Stem Cell & Regenerative Medicine, Shenzhen 518122, China

**Keywords:** nanoplastics, chemical modification, neurotoxicity, transgenerational, nematode

## Abstract

Increasing evidence has suggested that nanoplastic pollution has become a global concern. More importantly, transgenerational toxicity can be induced by nanoplastics at predicted environmentally relevant doses (ERDs). Considering that amino modification could increase nanoplastic toxicity, we compared transgenerational neurotoxicity between pristine polystyrene nanoparticle (PS-NP) and amino-modified PS-NP (NH_2_-PS-NP) in *Caenorhabditis elegans*. At 0.1–10 μg/L, NH_2_-PS-NP caused more severe transgenerational toxicity on locomotion and neuronal development. Accompanied with a difference in transgenerational neuronal damage, compared to PS-NP (10 μg/L), NH_2_-PS-NP (10 μg/L) induced more severe transgenerational activation of *mec-4*, *crt-1*, *itr-1*, and *tra-3*, which are required for the induction of neurodegeneration. Moreover, NH_2_-PS-NP (10 μg/L) caused more severe transgenerational inhibition in expressions of *mpk-1*, *jnk-1*, *dbl-1*, and *daf-7* than PS-NP (10 μg/L), and RNA interference (RNAi) of these genes conferred susceptibility to the toxicity of PS-NP and NH_2_-PS-NP on locomotion and neuronal development. NH_2_-PS-NP (10 μg/L) further caused more severe transgenerational activation of germline ligand genes (*ins-3*, *ins-39*, *daf-28*, *lin-44*, *egl-17*, *efn-3*, and *lag-2*) than PS-NP (10 μg/L), and RNAi of these ligand genes caused resistance to the toxicity of PS-NP and NH_2_-PS-NP on locomotion and neuronal development. Our results highlighted more severe exposure risk of amino-modified nanoplastics at ERDs in causing transgenerational neurotoxicity in organisms.

## 1. Introduction

Together with the increased generation of waste plastics, their ecological risk has also been assessed and received attention [1,2]. This is largely due to the environmental existence of microplastics and nanoplastics caused by release after human use or the fragmentation of waste plastics undergoing degradation [3,4]. Nanoplastics are distributed ubiquitously in the environment encompassing marine and aquatic environments [5,6], and detected in the tissues of organisms and in the food web [7,8]. Accompanied with body accumulation, nanoplastic exposure leads to multiple toxicities in organisms, such as reproductive impairment and damage to organ systems [9,10,11]. The predicted environmentally relevant doses (ERDs) range in ng/L or μg/L for nanoplastics [12,13]. For example, nanoplastics could be detected in sampled Swedish lakes and streams at mean doses of 563 μg/L [14]. Nanoplastics at ERDs could further induce some toxic effects on both plants and animals, such as the induction of oxidative damage and ferroptosis [15,16,17,18]. Moreover, nanoplastics caused transgenerational toxicity in the offspring of exposed organisms, such as rotifers and fish [19,20,21,22].

*Caenorhabditis elegans* exhibits high sensitivity to environmental pollutants [23,24,25,26]. *C. elegans* is thus helpful to detect pollutant toxicity at ERDs [27,28,29,30,31]. It can be applied for the toxicological study of both microplastics and nanoplastics in several aspects, such as reproductive toxicology [32,33]. During development, the life cycle of *C. elegans* is only approximately 4–5 days, and this makes it suitable to assess the transgenerational toxicity of a pollutant [34,35,36]. Pristine and aged polystyrene nanoparticles (PS-NPs) at ERDs resulted in transgenerational damage to the functions of neurons and gonads [37,38]. Transgenerational PS-NP toxicity is regulated by some secreted ligands, including Notch and fibroblast growth factor (FGF) ligands [39,40]. Additionally, epigenetic regulations, such as histone methylation, also control the transgenerational toxicity of PS-NPs [41,42,43].

The nanoplastic toxicity induction was influenced by some determining factors, including sizes, type, and source [44,45]. Besides these, in the parental generation (P0-G), PS-NP toxicity was also influenced by some chemical modifications, including amino modification and epoxy modification [46,47]. For example, amino modification strengthened PS-NP toxicity in P0-G [48]. However, it is not entirely clear why this modification is thought to have this impact. Among the sublethal endpoints used for toxicity assessment, the endpoints reflecting neurotoxicity showed a more sensitive property in nematodes [49]. We assumed that amino-modified PS-NPs at ERDs may induce more severe transgenerational neurotoxicity compared to pristine PS-NPs. Thus, we aimed to compare transgenerational neurotoxicity between pristine and amino-modified PS-NPs. The neurotoxicity of pollutants is reflected by the damage on both the development and function of *C. elegans* neurons [50]. Locomotion is controlled by the motor neurons in GABAergic neurons [51], and the motor neurons could be damaged by PS-NPs in P0-G [52]. Moreover, some molecular signals (DBL-1, DAF-7, JNK-1, and MPK-1) functioned in neurons to control PS-NP toxicity in P0-G [53,54,55,56]. We further determined the underlying mechanism for possible enhancement in transgenerational PS-NP neurotoxicity by amino modification. The results suggest that exposure to amino-modified nanoplastics carries a more severe risk for causing transgenerational neurotoxicity.

## 2. Materials and Methods

### 2.1. Nanoplastic Properties

**Figure 1:**
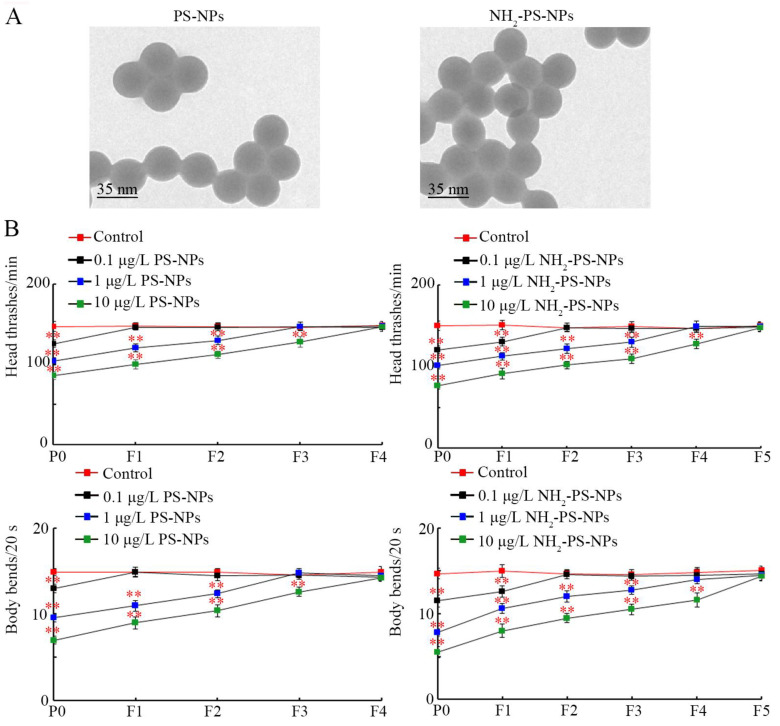
Comparison of transgenerational effect between pristine and amino-modified PS-NPs on locomotion behavior. (**A**) TEM images of pristine and amino-modified PS-NPs before sonication. (**B**) Comparison of transgenerational effect between pristine and amino-modified PS-NPs on head thrash and body bend. ** *p* < 0.01 vs. control.

The pristine PS-NPs (35 nm) and amino-modified PS-NPs (NH_2_-PS-NP, 35 nm) were gifts from Prof. Xianzheng Yuan’s lab [48]. Other reagents were purchased from Sigma-Aldrich (Milwaukee, Germany). The morphology was spherical, and the particle sizes were 34.7 ± 3.6 nm (PS-NPs) and 35.2 ± 3.1 nm (NH_2_-PS-NPs, 35 nm), respectively, confirmed by transmission electron microscopy (Figure 1A). The zeta potentials of the PS-NPs and NH_2_-PS-NPs were −19.8 ± 1.79 mV and −25.1 ± 0.87 mV, respectively. The FTIR spectrum and the Raman spectrum of the PS-NPs and NH_2_-PS-NPs have been described in our previous report [48].

### 2.2. Animal Maintenance

Wild-type N2 from the Caenorhabditis Genetics Center was grown on nematode growth medium (NGM) plates, and *Escherichia coli* OP50 was fed as *C. elegans* food [57]. When adults attained maximum oviposition, they were lysed with a lysis solution (2% HOCl, 0.45 M NaOH). The eggs were placed onto an NGM medium to grow into L1 larvae [58]. *C. elegans* was cultured in strict accordance with the ARRIVE Guidelines.

### 2.3. Exposure

Concentrations of PS-NPs (0.1–10 μg/L) were selected [59], which belong to the predicted ERDs of the nanoplastics [12,13,14]. The *C. elegans* were placed in a solution containing PS-NPs from L1 larvae for 6.5 days, referred to as P0-G. During exposure, the PS-NPs were replaced daily. The eggs of the P0-G were transferred to NGM plates to develop into adulthood, referred to as F1-G. The following generations of offspring (F2-G to Fn-G) were also prepared in the same way.

### 2.4. Neurotoxicity Assessment

Locomotion reflects the function of the motor neurons [60]. To assess the effect on locomotion, animals were allowed to recover for one minute before assessing their head thrashes and body bends. A head thrash is defined as a change in the direction of head movement [61], and a body bend is defined as a change in direction at mid-body [62]. Fifty animals were analyzed for each treatment.

A transgenic strain of EG1285 with the fused expression of GFP was used to visualize the D-type motor neurons [63]. The extent of neurotoxicity by PS-NPs was reflected by the number of neurons, ventral cord gap, fluorescence intensity, and cell body size of neurons [64]. The cell body size and GFP fluorescence intensity were semi-quantified using Image J software. The number of neurons and the ventral cord gap on the ventral cord were directly counted under a laser confocal microscope. Fifty animals were analyzed for each treatment.

### 2.5. Gene Expression

The nematodes were added to Trizol reagent to extract their RNA and kept at −80 °C. The cDNA was synthesized next. A quantitative real-time polymerase chain reaction (qRT-PCR) was conducted with the SYBR Green PCR kit (Takara, Kusatsu, Japan). The PCR cycling conditions were an initial denaturation at 95 °C for 5 min, followed by 32 cycles of 94 °C for 30 s, 52 °C for 30 s, and extension at 72 °C for 30 s. *tba-1* acted as the reference gene for the normalization of the target genes [65]. Information on the primers is provided in Table S1. Three replicates were performed.

### 2.6. RNA Interference (RNAi)

RNAi constructs with gene-specific sequences were transforming into *E. coli* HT115 [66]. The RNAi bacteria were cultured in LA medium overnight, followed by treatment with 100 μg/mL tetracycline and 5 mM isopropyl thiogalactoside for 5 h [67]. RNAi was generated by feeding the L1 larvae with RNAi bacteria. The offspring were exposed to PS-NPs. L4440, an empty vector, acted as the control [68]. The RNAi efficiency was confirmed by qRT-PCR (Figure S1).

### 2.7. Data Analysis

Data are presented as means ± standard derivation (SD). Statistical analysis was conducted by SPSS v19.0 software (IBM, Armonk, NY, USA). The significant difference among different groups was examined using one-way or two-way analysis of variance (ANOVA) followed by the Tukey post hoc test. The *p*-value of <0.01 (**) was deemed statistically significant.

## 3. Results

### 3.1. Amino Modification Increased Transgenerational Toxicity of PS-NPs on Locomotion

After exposure in P0-G, 0.1 μg/L PS-NPs did not cause toxicity on locomotion in the offspring, whereas 1 μg/L PS-NPs caused a decrease in locomotion from P0-G to F2-G, and 10 μg/L PS-NPs induced a decrease in locomotion from P0-G to F3-G (Figure 1B). Different from these, after P0-G exposure, 0.1 μg/L NH_2_-PS-NPs could affect locomotion in both P0-G and F1-G; 1 μg/L NH_2_-PS-NPs altered locomotion from P0-G to F3-G; and 10 μg/L NH_2_-PS-NPs decreased locomotion from P0-G to F4-G (Figure 1B).

### 3.2. Amino Modification Increased Transgenerational Toxicity of PS-NPs on Neuronal Development of D-Type Motor Neurons

**Figure 2:**
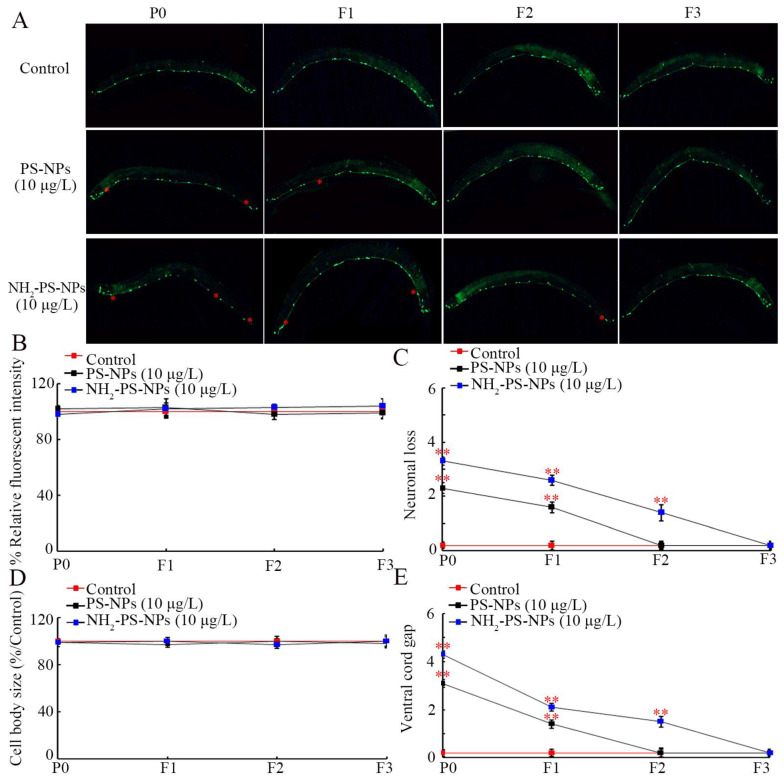
Comparison of transgenerational effect between pristine and amino-modified PS-NPs on development of D-type motor neurons. (**A**) Images of D-type motor neurons. Transgenic strain of EG1285 with the fused expression of GFP was used to visualize D-type motor neurons. The asterisks indicate the position with neuronal loss. (**B**) Comparison of relative fluorescence intensity. (**C**) Comparison of neuronal loss. (**D**) Comparison of cell body size. (**E**) Comparison of ventral cord gaps. ** *p* < 0.01 vs. control.

Motor neurons are located on the ventral nerve cord of the *C. elegans* GABAergic system. Exposure to 10 μg/L PS-NPs and NH_2_-PS-NPs all did not affect the fluorescent intensity and size of the cell body of motor neurons in P0-G and in their offspring (Figure 2A–C). However, 10 μg/L PS-NPs resulted in neuronal loss and ventral cord gap in both P0-G and F1-G (Figure 2C,E). Moreover, 10 μg/L NH_2_-PS-NPs caused neuronal loss and ventral cord gap from P0-G to F2-G (Figure 2C,E).

### 3.3. Amino Modification Strengthened Transgenerational Effect of PS-NPs on Expressions of Genes Governing Neurodegeneration

**Figure 3:**
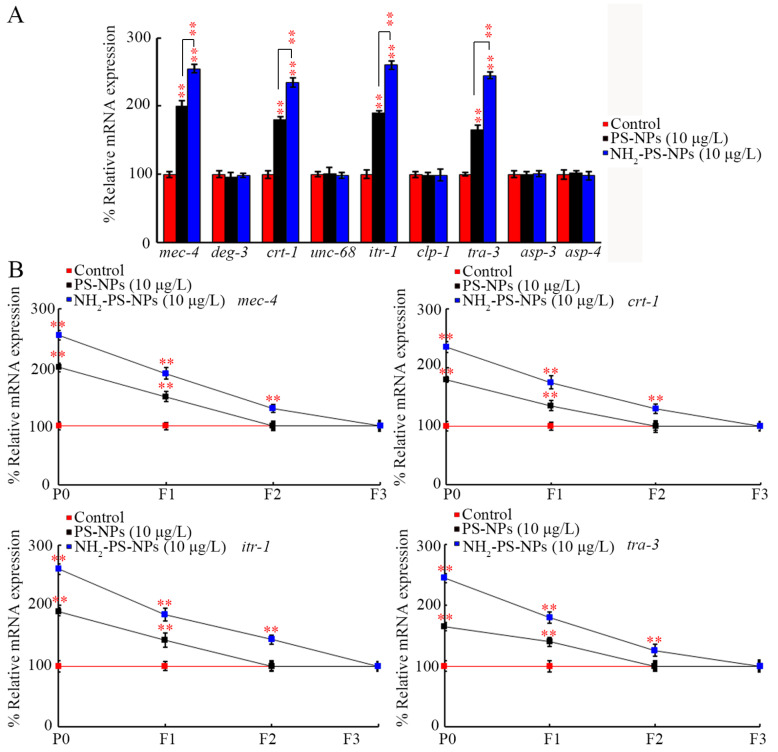
Comparison of transgenerational effect between pristine and amino-modified PS-NPs on expressions of genes governing neurodegeneration. (**A**) Comparison of effect between pristine and amino-modified PS-NPs on expressions of genes governing neurodegeneration at P0-G. (**B**) Comparison of transgenerational effect between pristine and amino-modified PS-NPs on expressions of *mec-4*, *ctr-1*, *itr-1*, and *tra-3*. ** *p* < 0.01 vs. control (if not specially indicated).

To determine the molecular basis for the difference between pristine and amino-modified PS-NPs in causing transgenerational toxicity on neuronal development, we compared transgenerational expressions of the genes governing neurodegeneration. Among the examined genes, PS-NPs (10 μg/L) and NH_2_-PS-NPs (10 μg/L) did not affect the expressions of *deg-3*, *unc-68*, *clp-1*, *asp-3*, and *asp-4* in P0-G (Figure 3A). However, the expressions of *mec-4*, *ctr-1*, *itr-1*, and *tra-3* were increased by PS-NPs (10 μg/L) and NH_2_-PS-NPs (10 μg/L), and NH_2_-PS-NPs (10 μg/L) caused a more severe increase in the expressions of *mec-4*, *ctr-1*, *itr-1*, and *tra-3* compared to those in PS-NP (10 μg/L)-exposed nematodes in P0-G (Figure 3A).

After PS-NP (10 μg/L) exposure, increased expressions of *mec-4*, *ctr-1*, *itr-1*, and *tra-3* were detected in F1-G (Figure 3B). Different from this, after NH_2_-PS-NP exposure, increased expressions of *mec-4*, *ctr-1*, *itr-1*, and *tra-3* were observed in F1-G and F2-G (Figure 3B).

### 3.4. Amino Modification Strengthened Transgenerational Inhibition of jnk-1, mpk-1, daf-7, and dbl-1 by PS-NP Exposure

**Figure 4:**
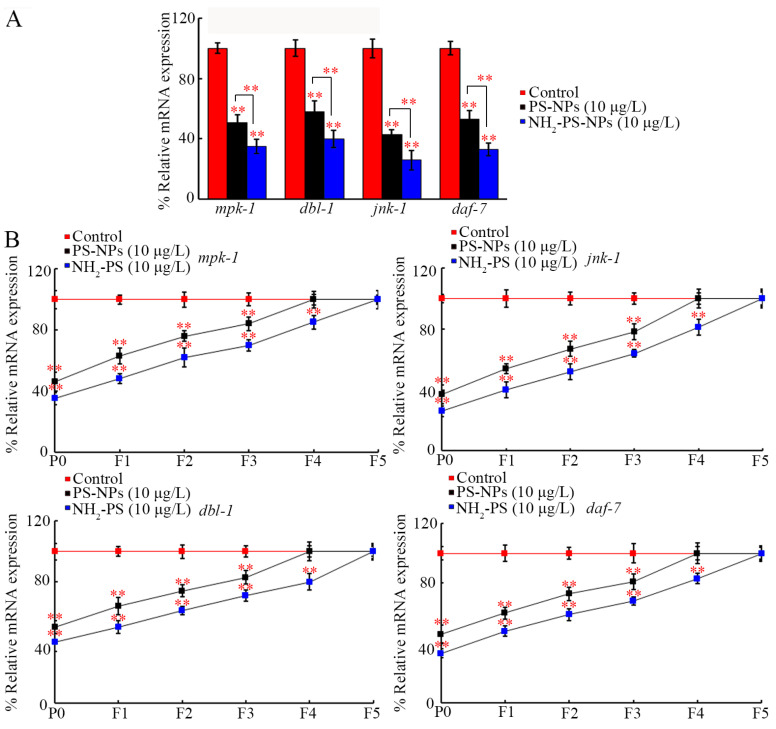
Comparison of transgenerational effect between pristine and amino-modified PS-NPs on expressions of *mpk-1*, *dbl-1*, *jnk-1*, and *daf-7*. (**A**) Comparison of effect between pristine and amino-modified PS-NPs on expressions of *mpk-1*, *dbl-1*, *jnk-1*, and *daf-7* at P0-G. (**B**) Comparison of transgenerational effect between pristine and amino-modified PS-NPs on expressions of *mpk-1*, *dbl-1*, *jnk-1*, and *daf-7*. ** *p* < 0.01 vs. control (if not specially indicated).

Considering the requirement of neuronal JNK-1, MPK-1, DAF-7, and DBL-1 for the toxicity of PS-NPs [53,54,55,56], we also compared the effect between pristine and amino-modified PS-NPs on their expressions. In P0-G, their expressions were decreased by PS-NPs (10 μg/L) and NH_2_-PS-NPs (10 μg/L), and NH_2_-PS-NPs (10 μg/L) caused a more severe inhibition in their expressions than PS-NPs (10 μg/L) did (Figure 4A).

After exposure to PS-NPs (10 μg/L) in P0-G, a decrease in these four genes’ expressions was also observed from F1-G to F3-G (Figure 4B). Different from this, after NH_2_-PS-NP (10 μg/L) exposure in P0-G, a decrease in their expressions could be further found from F1-G to F4-G (Figure 4B).

### 3.5. RNAi of jnk-1, mpk-1, daf-7, and dbl-1 Increased Neurotoxicity of Both Pristine and Amino-Modified PS-NPs

**Figure 5:**
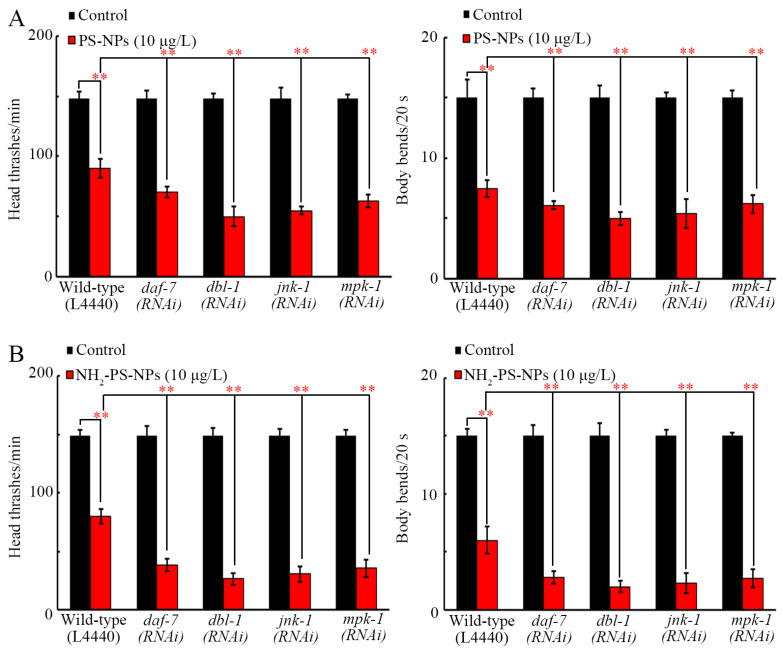
Effect of RNAi of *mpk-1*, *dbl-1*, *jnk-1*, and *daf-7* on toxicity of PS-NP (**A**) and NH_2_-PS-NP (**B**) in decreasing locomotion behavior. ** *p* < 0.01.

Under normal conditions, locomotion was not affected by RNAi of *jnk-1*, *mpk-1*, *daf-7*, and *dbl-1* (Figure 5A,B). After PS-NP exposure, more severe locomotory inhibition was observed in *jnk-1(RNAi)*, *mpk-1(RNAi)*, *daf-7(RNAi)*, and *dbl-1(RNAi)* nematodes than that in wild-type nematodes (Figure 5A). Similarly, RNAi of these genes caused a more severe inhibition in locomotion in NH_2_-PS-NP-exposed nematodes (Figure 5B). Meanwhile, RNAi of these genes resulted in a more severe induction of neurodegeneration reflected by the related endpoints in PS-NP- or NH_2_-PS-NP-exposed nematodes (Figure S2).

### 3.6. Amino Modification Strengthened Transgenerational Activation of Germline Ligand Genes by PS-NP Exposure

**Figure 6:**
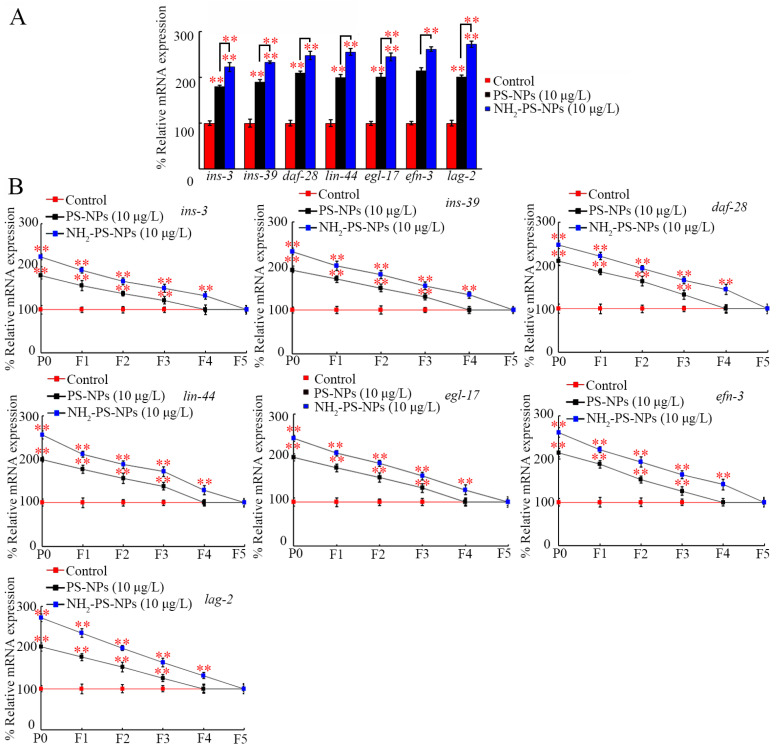
Comparison of transgenerational effect between pristine and amino-modified PS-NPs on expressions of *ins-3*, *ins-39*, *daf-28*, *lin-44*, *egl-17*, *efn-3*, and *lag-2*. (**A**) Comparison of effect between pristine and amino-modified PS-NPs on expressions of *ins-3*, *ins-39*, daf-28, *lin-44*, *egl-17*, *efn-3*, and *lag-2* at P0-G. (**B**) Comparison of transgenerational effect between pristine and amino-modified PS-NPs on expressions of *ins-3*, *ins-39*, *daf-28*, *lin-44*, *egl-17*, *efn-3*, and *lag-2*. ** *p* < 0.01 vs. control (if not specially indicated).

The germline ligands of insulin, Wnt, FGF, Ephrin, and Notch regulated the transgenerational PS-NP toxicity [39,40,59,69,70]. In P0-G, the germline expressions of *ins-3*, *ins-39*, *daf-28*, *lin-44*, *egl-17*, *efn-3*, and *lag-2* were increased by PS-NPs (10 μg/L) and NH_2_-PS-NPs (10 μg/L), and NH_2_-PS-NPs (10 μg/L) resulted in a more severe increase in the germline expressions of these ligand genes than PS-NPs (10 μg/L) did (Figure 6A).

After PS-NP (10 μg/L) exposure in P0-G, increased germline expressions of these ligand genes could be further found from F1-G to F3-G (Figure 6B). After exposure to NH_2_-PS-NPs (10 μg/L) in P0-G, activation of these germline ligand genes was further observed from F1-G to F4-G (Figure 6B).

### 3.7. RNAi of ins-3, ins-39, daf-28, lin-44, egl-17, efn-3, and lag-2 Inhibited Neurotoxicity of Both Pristine and Amino-Modified PS-NPs

**Figure 7:**
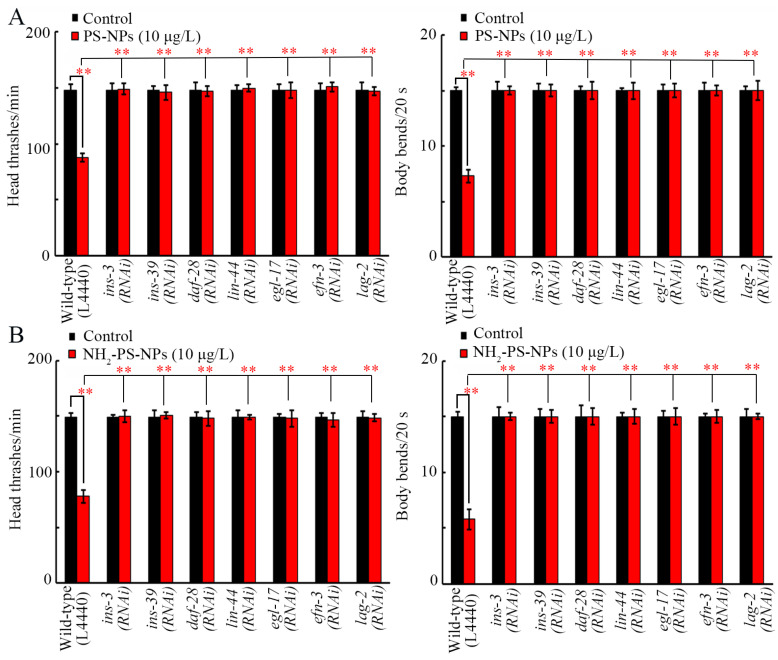
Effect of RNAi of *ins-3*, *ins-39*, *daf-28*, *lin-44*, *egl-17*, *efn-3*, and *lag-2* on toxicity of PS-NPs (**A**) and NH_2_-PS-NPs (**B**) in decreasing locomotion behavior. ** *p* < 0.01.

The decrease in locomotion caused by PS-NPs and NH_2_-PS-NPs could be significantly inhibited by RNAi of *ins-3*, *ins-39*, *daf-28*, *lin-44*, *egl-17*, *efn-3*, and *lag-2* (Figure 7A,B). Moreover, the neurodegeneration induced by PS-NPs and NH_2_-PS-NPs was also suppressed by RNAi of *ins-3*, *ins-39*, *daf-28*, *lin-44*, *egl-17*, *efn-3*, and *lag-2* (Figure S3).

## 4. Discussion

It has been well recognized that amino modification could increase the adverse effects of nanoplastics on organisms [47,71]. Amino modification increased both the cytotoxicity and genotoxicity of PS-NPs in A549 cells [71]. Amino modification further enhanced PS-NP toxicity on the reproductive system in male mice [47]. Amino modification could even affect the effect of PS-NPs on microbial communities in sediment [72]. In nematodes, amino modification increased PS-NP reproductive toxicity in P0-G [48]. In the current study, we further observed that amino modification strengthened the transgenerational PS-NP neurotoxicity on both locomotion and neuronal development. With 10 μg/L as the example, PS-NPs caused decreased locomotion behavior from P0-G to F3-G, whereas NH_2_-PS-NPs resulted in decreased locomotion behavior from P0-G to F4-G (Figure 1B). Meanwhile, compared to the neurodegeneration induced in P0-G and F1-G by PS-NPs (10 μg/L), NH_2_-PS-NPs (10 μg/L) caused neurodegeneration from P0-G to F2-G (Figure 2). These demonstrated the potential of amino modification in enhancing transgenerational PS-NP toxicity on both locomotion and neuronal development. Compared to the endpoints reflecting neuronal development, the endpoints reflecting locomotion were relatively more sensitive for assessing the transgenerational toxicity of nanoplastics. The reproductive toxicity of PS-NPs was also increased by amino modification, and a 4-day exposure of L1 larvae to NH_2_-PS-NPs (10 μg/L) induced inhibition in the brood size in both P0-G and F1-G [73]. Hormesis is an adaptative response induced by stresses and pollutants to protect biological systems against damage formation [74,75,76]. What we performed was long-term exposure to both 35 nm PS-NPs and 35 nm NH_2_-PS-NPs. Nevertheless, we did not observe the hormesis response after exposure to 35 nm PS-NPs and 35 nm NH_2_-PS-NPs. Different from this, we observed the hormesis response after exposure to 100 nm PS-NPs at similar doses [77,78,79,80].

The transgenerational damage of PS-NPs on the neuronal development of D-type motor neurons by amino modification was partially due to the differential effect on genes governing neurodegeneration. In *C. elegans*, the activation of DEF-3 and MEC-4, two excitotoxic-like ion channels, triggers neurodegeneration [81]. Both PS-NPs (10 μg/L) and NH_2_-PS-NPs (10 μg/L) could increase *mec-4* expression, and NH_2_-PS-NPs (10 μg/L) caused a more severe transgenerational increase in *mec-4* expression than PS-NPs (10 μg/L) did (Figure 3B). Endoplasmic reticulum (ER)-residential calcium chaperon CTR-1 and inositol 1,4,5-riphosphate receptor ITR-1 act downstream of MEC-4 to control calcium ion release from the ER [82,83]. Following MEC-4 activation, NH_2_-PS-NPs (10 μg/L) induced a more severe transgenerational increase in *ctr-1* and *itr-1* expressions than PS-NPs (10 μg/L) did (Figure 3B). Neurodegeneration is directly caused by the activation of the proteases, calpain proteases and aspartyl proteases [84]. Among the genes encoding these proteases, NH_2_-PS-NPs (10 μg/L) further caused a more severe transgenerational increase in the expression of *tra-3* encoding calpain protease than PS-NPs (10 μg/L) did (Figure 3B).

For an enhancement in the transgenerational PS-NP neurotoxicity, we raised two other aspects of the molecular mechanisms. One of them was that amino modification enhanced the transgenerational PS-NP neurotoxicity by causing more severe transgenerational inhibition in MPK-1, JNK-1, DBL-1, and DAF-7. On the one hand, NH_2_-PS-NPs (10 μg/L) caused a more severe transgenerational decrease in their expressions than PS-NPs (10 μg/L) did (Figure 4B). On the other hand, susceptibility to the toxicity of PS-NPs and NH_2_-PS-NPs on D-type motor neurons involved in locomotion and development was caused by RNAi of these genes (Figure 5 and Figure S2). In *C. elegans*, MPK-1, JNK-1, DBL-1, and DAF-7 functioned in neurons to regulate nanoplastic toxicity [53,54,55,56]. Therefore, the transgenerational inhibition of these neuronal signals mediated the toxicity of PS-NPs and NH_2_-PS-NPs across multiple generations.

Besides the role of these neuronal signals, the transgenerational activation of germline secreted ligands also contributed to the toxicity induction of PS-NPs and NH_2_-PS-NPs across multiple generations. NH_2_-PS-NPs (10 μg/L) caused a more severe transgenerational increase in the expressions of germline insulin, Wnt, FGF, Ephrin, and Notch ligand genes than PS-NP (10 μg/L) did (Figure 6B). Moreover, resistance to the neurotoxicity of PS-NPs and NH_2_-PS-NPs could be induced by RNAi of insulin, Wnt, FGF, Ephrin, and Notch ligand genes (Figure 7 and Figure S3). In *C. elegans*, germline INS-3, INS-39, DAF-28, LIN-44, EGL-17, EFN-3, and LAG-2 functioned together with their receptors (DAF-2, MIG-1, EGL-15, VAB-1, and GLP-1) to regulate the transgenerational nanoplastic toxicity [39,40,59,69,70]. That is, amino modification could further enhance transgenerational PS-NP neurotoxicity by resulting in a more severe transgenerational activation of germline insulin, Wnt, FGF, Ephrin, and Notch ligand genes.

## 5. Conclusions

Together, NH_2_-PS-NPs could cause more severe transgenerational neurotoxicity than PS-NPs at 0.1–10 μg/L in *C. elegans*. The observation of more severe transgenerational damage on D-type motor neurons by NH_2_-PS-NPs than PS-NPs was partially due to the more severe transgenerational activation of genes governing neurodegeneration. NH_2_-PS-NPs also caused more severe transgenerational neurotoxicity than PS-NPs by inducing more severe transgenerational inhibition in the expressions of *mpk-1*, *jnk-1*, *dbl-1*, and *daf-7*, and transgenerational activation of the expressions of germline ligand genes (*ins-3*, *ins-39*, *daf-28*, *lin-44*, *egl-17*, *efn-3*, and *lag-2*). Compared with the well-described role of amino modification in increasing the nanoplastic toxicity in P0-G, our results provided an important molecular basis for amino modification to enhance the transgenerational neurotoxicity of PS-NPs. Our data further implied that the exposure risk of amino-modified nanoplastics in causing more severe transgenerational toxicity needs to be carefully paid attention to. In the future, neuroprotective compounds (such as natural extracts or drugs) with the function of attenuating or preventing transgenerational neurotoxicity induced by PS-NPs and NH_2_-PS-NPs by upregulating MPK-1, JNK-1, DBL-1, and DAF-7 are suggested to be further screened and identified using *C. elegans* as an animal model.

## Data Availability

The original data presented in the study are included in the article/Supplementary Material; further inquiries can be directed to the corresponding authors.

## Supplementary Materials

The following supporting information can be downloaded at https://www.mdpi.com/article/10.3390/toxics12080555/s1: Figure S1: RNAi efficiency of *daf-7*, *jnk-1*, *dbl-1*, and *mpk-1*; Figure S2: Effect of RNAi of *mpk-1*, *dbl-1*, *jnk-1*, and *daf-7* on toxicity of PS-NP and NH2-PS-NP in causing damage on D-type motor neurons on GABAergic system; Figure S3: Effect of RNAi of *ins-3*, *ins-39*, *daf-28*, *lin-44*, *egl-17*, *efn-3*, and *lag-2* on toxicity of PS-NP and NH2-PS-NP in causing damage on D-type motor neurons on GABAergic system; Table S1: Primer information for qRT-PCR.
