# Supplementary material for: Comparison of Transgenerational Neurotoxicity between Pristine and Amino-Modified Nanoplastics in C. elegans
Source: Toxics. 2024 Jul 30;12(8):555. doi: 10.3390/toxics12080555 (PMC11358997; doi:10.3390/toxics12080555)
Supplement: Supplementary file 1 [file toxics-12-00555-s001.zip › toxics-3100212-supplementary.pdf]

Supporting Information:

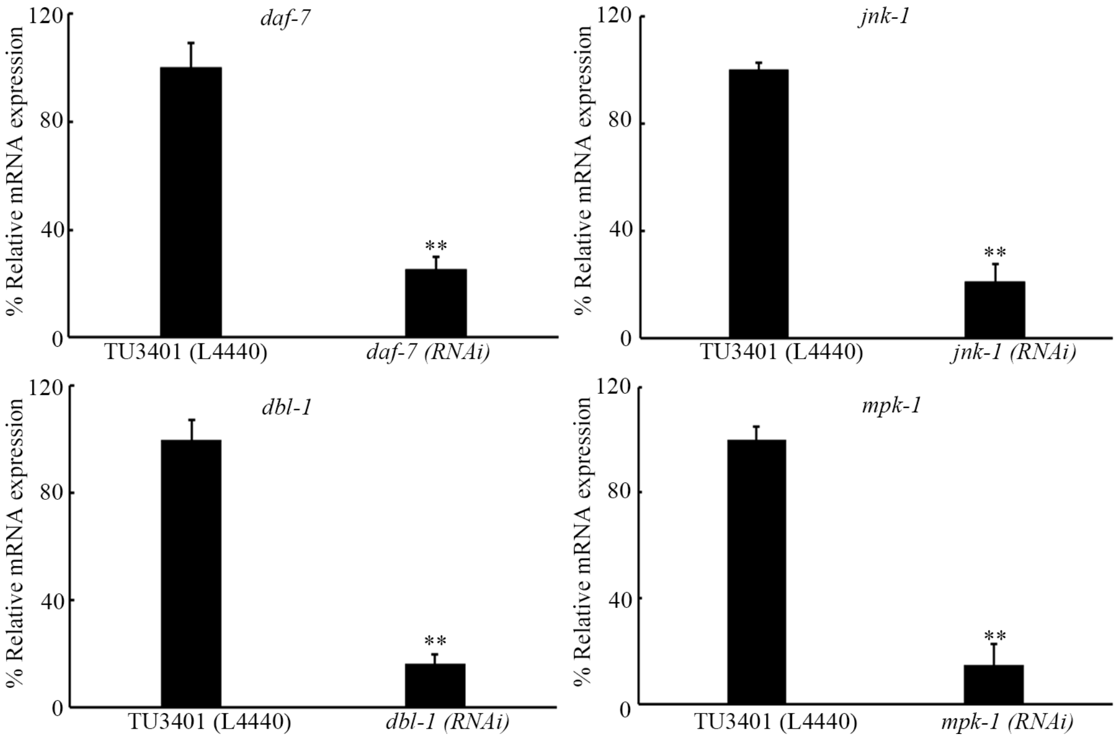

Fig. S1 RNAi efficiency of *daf-7*, *jnk-1*, *dbl-1*, and *mpk-1*. $^{**}P < 0.01$ *vs* wild-type(L4440).

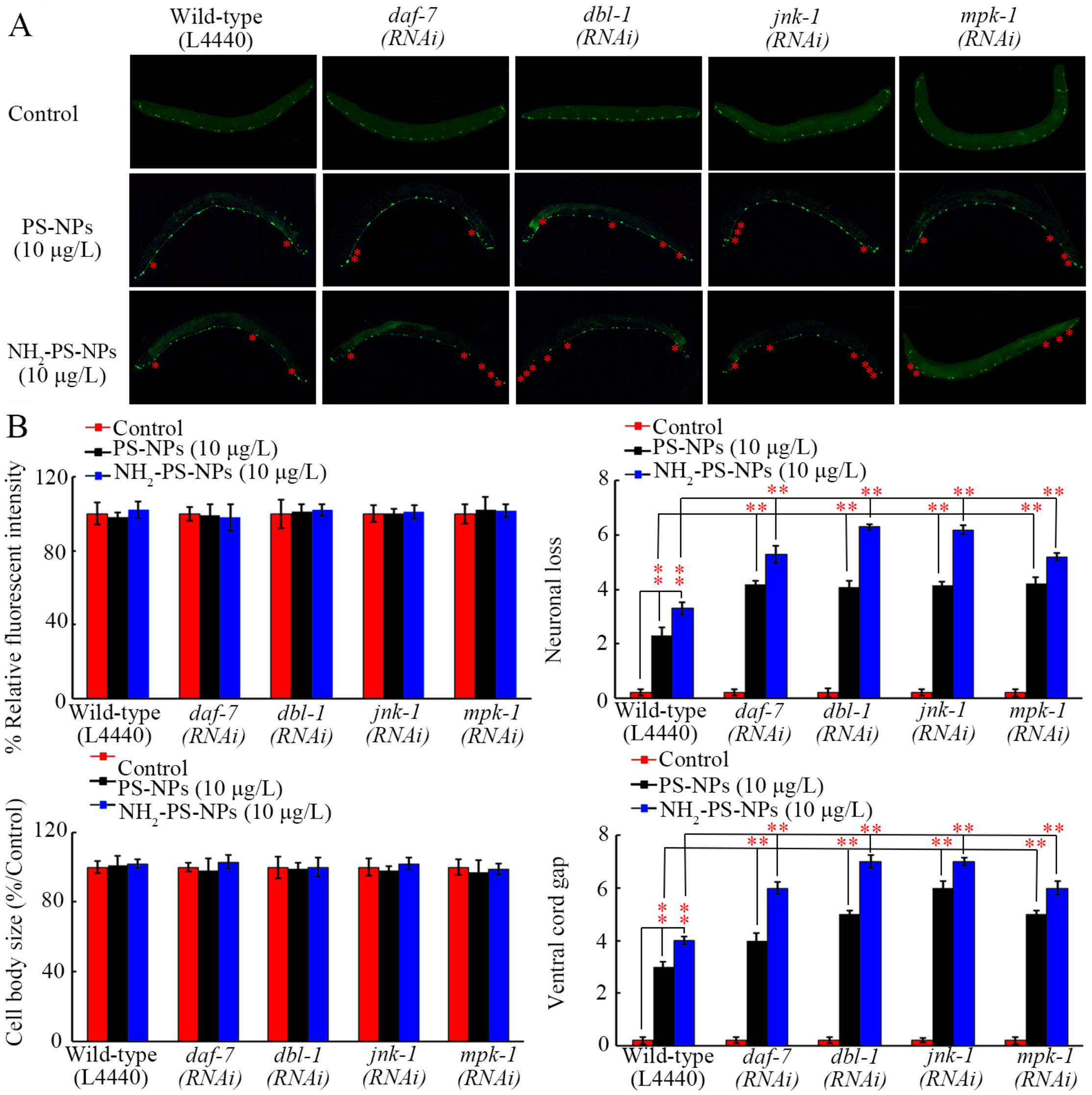

Fig. S2 Effect of RNAi of *mpk-1*, *dbl-1*, *jnk-1*, and *daf-7* on toxicity of PS-NP and NH_2-PS-NP in causing damage on D-type motor neurons on GABAergic system. $^{**}P < 0.01$.

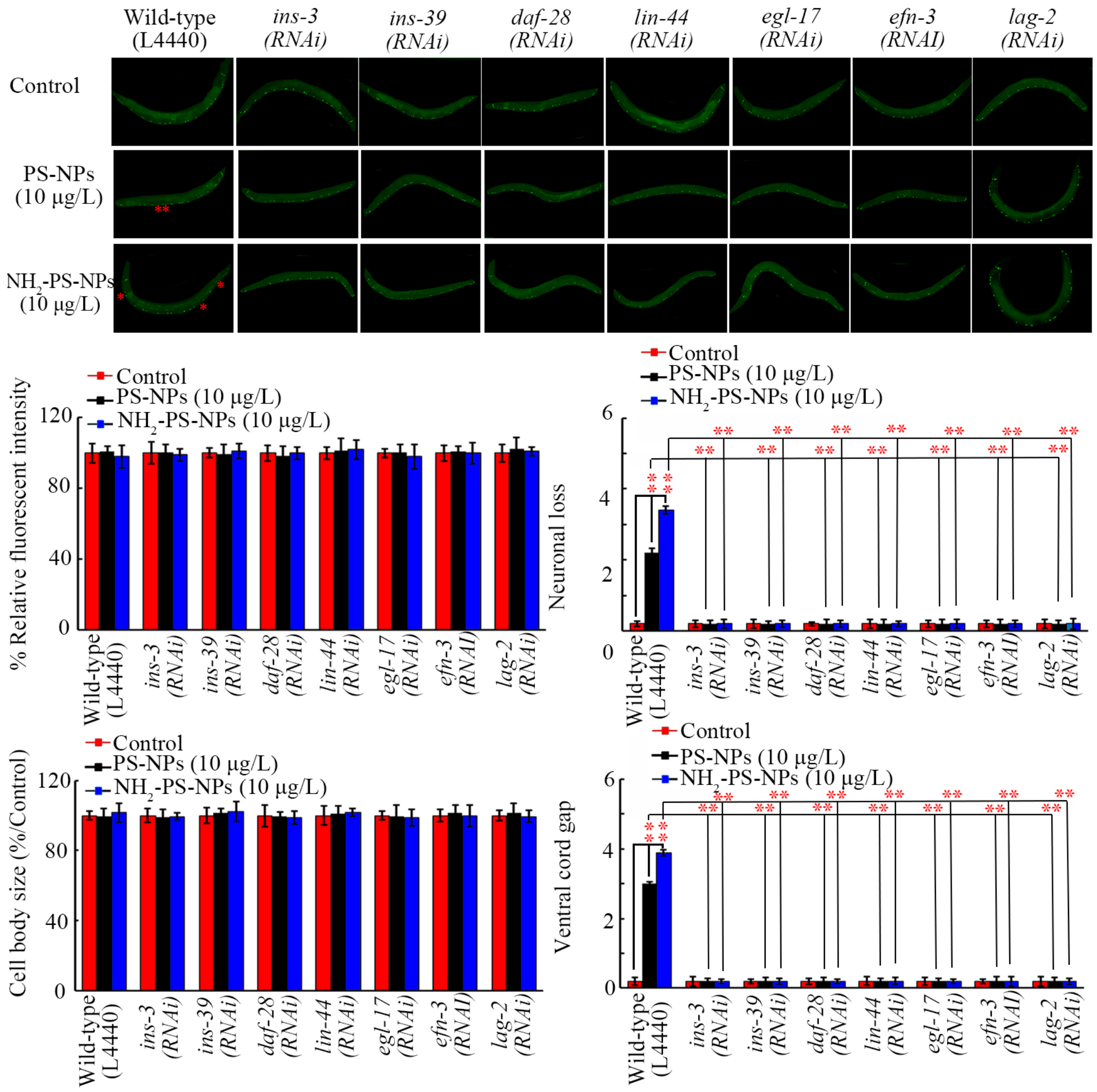

Fig. S3 Effect of RNAi of *ins-3*, *ins-39*, *daf-28*, *lin-44*, *egl-17*, *efn-3*, and *lag-2* on toxicity of PS-NP and NH_2-PS-NP in causing damage on D-type motor neurons on GABAergic system. $^{**}P < 0.01$.

Table S1. Primer information for qRT-PCR

Gene	Forward primer (5’-3’)	Reverse primer (5’-3’)
mec-4	CACGTTTCCCAGTCCCTGAA	CCACCATTGCTCCGTTCTCT
deg-3	TCTTGCTCCATCAACTGCCA	CGATGACGACTGCTCCTACG
unc-68	TGTTTCGGGTCAACTCAATGT	GGTTCGCTAAGGCACGTTTG
clp-1	TTTCACAGCAAAAGCCGACG	GTTGATCTCCGCTGTTCCCA
crt-1	CTGGGATGACGAGATGGACG	TCGTTCCTGACTTGACCTGC
itr-1	GCTCTTGCGATACCGAGTCA	AAGACCCATGCCTTCGAACA
tra-3	GTCCGAAAAGACCCGTCACT	TGACATCATTTGGCGAGGCT
asp-3	CCAAGGATTGGCCTGTGCTA	CCAAGGATTGGCCTGTGCTA
asp-4	CGTCAGGATGAACCGCTGTA	ATCCATGACGATGCTTGGCA
daf-7	CCCTTCATCCCCAACAGACC	GACATTGGCGATTGAGACGC
jnk-1	GCGCAGATGGTTGTTCTCAA	AAGCCCTCTTTGCATGTGTC
dbl-1	TTATGGCACCCAAGGGCTAC	TCAGTAGGCACACAGCAAGG
mpk-1	TGGAGAAGGTGCTTACGG	TTTGAGACCACGGAGAAT
ins-3	TCTTATGCGTAACTGGAT	CAGGGAGTGAATGTTATG
ins-39	TTGCAGCCGACGAGTTTC	CCCGACGTGTTTGATGGT
daf-28	TCATCGCCATCTTTGCCGTA	AAGAAGCAAACGTGGGCAAC
lin-44	ACCCTTGAGCACATTACCGA	AGCCGATCACAATCACCTTG
egl-17	GGTTTAATGGAAGCGACGCC	TTTGGGGGAGTTGAGCAGAC
efn-3	TTCCCGGTTGCGTTCAAAAC	CCATACCGTCAGGGATTGGG
lag-2	GACATCGGATGGATGGGACC	GGCGTCTTTGACACTGCAAG
tba-1	TCAACACTGCCATCGCCGCC	TCCAAGCGAGACCAGGCTTCAG
